# Surface Modification with NGF-Loaded Chitosan/Heparin Nanoparticles for Improving Biocompatibility of Cardiovascular Stent

**DOI:** 10.1155/2021/9941143

**Published:** 2021-04-24

**Authors:** Haixin Song, Tao Wu, Xiaotian Yang, Yangzheng Li, Ye Ye, Bo Li, Tao Liu, Shihui Liu, Jianhua Li

**Affiliations:** ^1^Department of Rehabilitation, Sir Run Run Shaw Hospital, College of Medicine, Zhejiang University, Hangzhou, 310000 Zhejiang Province, China; ^2^Department of Rehabilitation, Tongde Hospital of Zhejiang Province, Hangzhou, 310000 Zhejiang Province, China; ^3^Medical College of Acu-Moxi and Rehabilitation, Guangzhou University of Chinese Medicine, Guangzhou, 510006 Guangdong Province, China

## Abstract

Late thrombosis and restenosis remain major challenges to the safety of drug-eluting stents. Biofunctional modification to endow the surface with selective anticoagulation and promote endothelium regeneration has become a hotpot recently. In this study, chitosan and heparin were found to form three-dimensional nanoparticles by spontaneous electrostatic interaction. Based on the specific binding properties between heparin and nerve growth factor (NGF), a new type of NGF-loaded heparin/chitosan nanoparticles was constructed for surface modification. The results of material characterization show that the nanoparticles are successfully immobilized on the surface of the material. In vitro blood compatibility and endothelial cell compatibility assay showed that the modified surface could selectively inhibit platelet adhesion and smooth muscle cell overproliferation, while accelerating endothelialization via promoting endothelial cell proliferation and enhancing endothelial progenitor cell mobilization.

## 1. Introduction

Percutaneous coronary intervention by using drug-eluting stent (DES) system is the main method for the treatment of cardiovascular diseases. Commonly, DES is mainly composed of metal stents, antiproliferative drugs (such as rapamycin and paclitaxel), and polymer coatings. However, due to the insufficient biocompatibility of polymers and the nonspecific effects of antiproliferative drugs, delayed endothelial healing and chronic inflammation may occur after stent implantation and raise the risk of late in-stent thrombus and restenosis [1]. In this regard, the researchers suggest that the biofunctional modification of the vascular stent surface may contribute to improve the biocompatibility and reduce the incidence of postoperative complications.

Vascular endothelium is the natural barrier between blood and vascular tissue, which mediated the metabolic exchange between blood and tissue and can synthesize a variety of bioactive substances to ensure the normal contraction and relaxation of blood vessels as well as inhibit blood coagulation and maintain the biological function of smooth muscle cells [2, 3]. Therefore, it is widely accepted that the rapid formation of a complete endothelium layer on stent surface will be an ideal way to reduce the risk of complications after implantation [4]. Since the discovery of endothelial progenitor cells (EPCs), the mechanism of regeneration and healing after vascular endothelium injury has been redefined as the combined action of EC proliferation and migration from the surrounding vascular tissue and EPC mobilization and homing from the bone marrow. The process of endothelialization *in vivo* largely depends on the ordered synergistic effect of cytokines and extracellular matrix proteins on ECs and EPCs [5, 6]. Hence, various cytokines and adhesive proteins including VEGF [7], SDF-1 [8], laminin [9], collagen [10], and fibronectin [11] were incorporated into material surface to accelerate endothelialization. However, as bioactive macromolecules, proteins and cytokines have a short half-life *in vivo* and how to prevent the rapid inactivation of biological macromolecules *in vivo* continues to be problematic and needs to be solved urgently.

In recent years, multifunctional nanoparticle systems have shown great potential in controlling the release of biomolecules and regulating the behavior of vascular cells. Compared with other controlled release systems, the unique nanoeffect of the nanoparticle system greatly increases its drug loading capacity and can separate the active molecules from the surrounding environment to avoid rapid deactivation. In this regard, the researchers proposed to build biofunctional nanocoating with 3D structure on the surface of cardiovascular materials to enhance the biological activity and prolong the action duration of biomolecules, so as to achieve long-term and effective regulation of intravascular biological reactions [12]. Zhou et al. [13] covalently immobilized VEGF-loaded polycaprolactone (PCL) nanoparticles on acellular scaffolds and found that the nanocoating could significantly reduce the sudden release of biomolecules and promote the endothelialization of the material surface. Mohammadi et al. [14] introduced heparin/chitosan nanoparticles on the surface of anodized NiTi alloy and proved that the nanocoating can improve the surface blood compatibility and endothelial cell compatibility through controlled biomolecule release. In our previous study, a novel kind of heparin/poly-l-lysine nanoparticles was constructed for stent surface modification. We proved that this kind of nanocoating can effectively inhibit blood coagulation and reduce the occurrence of restenosis [15, 16]. However, some defects such as poor endothelial cell compatibility and significant cytotoxicity of high molecular weight poly-l-lysine continue to be resolved.

In this study, a new type of heparin/chitosan nanoparticles loaded with nerve growth factor (NGF) was designed for surface modification of cardiovascular materials. Chitosan (CHS) is a kind of natural polycationic polysaccharide, which has excellent antibacterial functions, promoting injury repair and reducing inflammatory reaction. In addition, CHS is also one of the most commonly used drug carriers due to its remarkable advantages such as biocompatibility, biodegradability, and easy chemical modification characteristics [17, 18]. NGF is an important neurotrophic factor, which plays an important role in maintaining the normal function of vascular endothelial tissue [19]. Studies have shown that NGF can promote the mobilization and homing of vascular endothelial progenitor cells (EPCs), showing the potential to accelerate the repair of vascular endothelial injury [20, 21]. NGF is also a heparin-binding protein; in this study, NGF was loaded into heparin/chitosan nanoparticles via the specific interaction between NGF and heparin to improve the cellular compatibility and accelerate endothelialization. According to a series of material characterization, *in vitro* blood compatibility and cellular compatibility evaluation, this study has proved that the surface modification of nanoparticles can effectively improve the anticoagulant properties of materials and accelerate endothelium regeneration.

## 2. Materials and Methods

### 2.1. Materials and Reagents

316L stainless steel (316L SS) was processed into round shape (*Φ* 10 mm, ~1.2 mm in thickness) and mirror polished. Dopamine (DA), toluidine blue O (TBO), and heparin (Hep) were purchased from Sigma-Aldrich. Chitosan (CHS, deacetylation ≥ 95%), rhodamine 123, and alcian blue were purchased from Shanghai Aladdin BioChem Technology Co., LTD.

### 2.2. CHS/Hep Nanocoating Construction

Firstly, a certain amount of CHS and Hep powder was dissolved in 0.1 M acetic acid/sodium acetate buffer (pH = 5), respectively. Then, equal volume of CHS was added dropwisely to the Hep solution (concentration ratio 1 mg/ml : 7.5 mg/ml) under magnetic stirring at room temperature to obtain the CHS/Hep nanoparticle suspension. For CHS/Hep@NGF nanoparticle preparation, 15 mg/ml Hep was firstly mixed with equal volume of 200 ng/ml NGF solution to obtain Hep@NGF mixture. The polydopamine coating was prepared on the surface of 316L SS (termed as SS-DA) according to the method described in our previous work [16]. After that, SS-DA was immersed in 0.5 ml CHS/Hep or CHS/Hep@NGF nanoparticle suspension and incubated at room temperature with gently shaking for 12 h to acquire the nanocoating (termed as SS-DA-NP or SS-DA-NP@NGF).

### 2.3. Size and Zeta Potential Assay

The mean size and particle dispersion index (PDI) as well as zeta potential of the prepared nanoparticles were determined by dynamic light scattering (DLS) using a ZETA-SIZER, MALVERN Nano-2S90 (Malvern Ltd., Malvern, UK).

### 2.4. FTIR and XPS Assay

Fourier transform infrared spectroscopy (FTIR) and X-ray photoelectron spectroscopy (XPS) were used to detect the alteration of the surface chemical structure and elemental composition during nanoparticle immobilization. The FTIR assay was performed on a Nicolet IS20 infrared spectrometer (Thermo, USA) using the model of attenuated total reflection. Infrared adsorption between 4000 and 650 cm^−1^ was recorded at room temperature. The XPS analysis was carried out by using VGESCALAB MK II spectrometer with a monochromatic Mg Ka X-ray source (1253.6 eV). The pressure of testing chamber was set as 8 × 10^−8^ Pa. The scale of binding energy was referenced by adjusting the C1s peak at 284.6 eV.

### 2.5. AFM Assay

The alteration of surface topography after NP modification was determined by atom force microscopy (AFM) (CSPM 6000, Being Nano-Instruments, China) in tapping model at room temperature with subsequent image analysis using CSPM Imager software. Before AFM analysis, the samples were rinsed twice with UP water and the surface was carefully blown dry.

### 2.6. Water Contact Angle Assay

The hydrophilicity of the samples was determined by measuring the static water contact angle using a Krüss GmbH DSA100 Mk 2 goniometer at room temperature. A droplet of UP water was added to the dried surface, and then, the contact angle was calculated by a circle segment function of the DSA 1.8 software. At least three different sites were taken into measurement for each sample.

### 2.7. Quantitative Characterization of Hep and Amine Group

The density of exposed amine group and heparin was determined by acid orange II (AO II) and TBO assay, respectively. Detailed method was according to our previous study [16].

### 2.8. In Vitro Blood Compatibility Evaluation

Fresh whole blood from a healthy volunteer was anticoagulated with sodium citrate (3.8 wt.%) at 9 : 1 and centrifuged at 1500 rpm for 15 min to obtain platelet-rich plasma (PRP). Next, 50 *μ*l PRP was added to each sample surface and incubated at 37°C for 2 h. Subsequently, the samples were rinsed gently with normal saline and used for morphology observation, lactate dehydrogenase (LDH) release, and P-selectin expression assay. Detailed method was according to our previous study [15].

### 2.9. In Vitro Cellular Compatibility Evaluation

#### 2.9.1. EC and SMC Proliferation Assay

Endothelial cells (ECs) were isolated from a human umbilical vein and cultured in DMEM/F12 medium containing 15% fetal bovine serum (FBS) and 20 *μ*g/ml endothelial cell growth supplement (ECGS). Smooth muscle cells (SMCs) were isolated from a human umbilical artery and cultured in DMEM/F12 medium (high glucose) containing 10% FBS. Before cell seeding, the dopamine-coated samples were sterilized by autoclaving, and the subsequent nanocoating construction was carried out under sterile conditions.

For cell proliferation assay, ECs or SMCs were seeded on the surface of each sample at the density of 5 × 10^4^ cells/cm^2^ and incubated at 37°C under 5% CO_2_ for 1 day and 3 days. At each time point, the supernatant was removed, and 500 *μ*l fresh culture medium containing 10% CCK-8 reagent was added and incubated for 4 h. Then, 200 *μ*l culture medium was transferred to a 96-well plate, and the absorbance was measured at 450 nm. The samples were gently rinsed with normal saline and fixed in 2.5% glutaraldehyde at room temperature for at least 6 h. Subsequently, 50 *μ*l rhodamine 123 solution was added to the surface of each sample and incubated at room temperature for 30 min. Finally, the samples were thoroughly rinsed with normal saline and the morphology of adherent ECs was observed by inverted fluorescent microscopy.

#### 2.9.2. EPC Mobilization and Homing

The mobilization and homing behavior of EPCs to the material surface induced by the nanoparticle coating was studied in vitro by transwell chamber method described by Liu et al. [22]. In detail, 3 ml fresh medium (*α*-MEM medium containing 10% FBS) was added to a 6-well plate, and then, 2 ml EPC suspension with the density of 5 × 10^5^ cells/ml was added to a 6-well Millipore transwell chamber with a bottom membrane pore diameter of 8 *μ*m. After incubation at 37°C for 2 hours, the sterilized samples with a size of 2 × 2 cm^2^ were immersed in the medium of 6-well plate and cultured at 37°C for 24 hours. After that, the transwell chamber was rinsed gently with normal saline and then fixed at room temperature in 90% ethanol solution for 30 minutes. The cells in the upper layer of the chamber were wiped with a wet cotton swab, and the chamber was placed in 0.1% crystal violet solution and stained at room temperature for 15 minutes. The cell mobilization in the lower layer of the chamber was observed under an inverted microscope.

### 2.10. Statistical Analysis

All the biological experiments were performed at least three times. The statistical data was analyzed using SPSS 22.0 software. Statistical evaluation of the data was performed using one-way ANOVA. The probability value *p* < 0.05 was considered significant.

## 3. Results and Discussion

### 3.1. Size and Zeta Potential of NPs

As shown in Table 1, the average particle size of CHS/Hep@NGF (133.4 ± 5.5 nm) was slightly decreased compared with that of CHS/Hep (147.8 ± 6.7 nm), which indicate that the incorporation of NGF may enhance the compactness of nanoparticles. The absolute value of zeta potential of both CHS/Hep and CHS/Hep@NGF was greater than 20 mV, indicating that the particle system had adequate stability to avoid the settlement caused by particle agglomeration. Besides, the PDI (particle dispersion index) of CHS/Hep and CHS/Hep@NGF was less than 0.2, indicating that the prepared nanoparticles have excellent size uniformity.

### 3.2. FTIR and XPS

**Figure 1:**
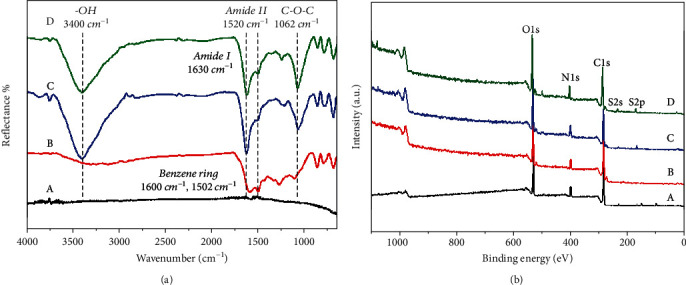
Surface chemical composition determined by (a) FTIR spectra and (b) XPS wide-scan spectra. (a)~(d) refer to 316L SS, SS-DA, SS-DA-NP, and SS-DA-NP@NGF, respectively.

In this paper, FTIR and XPS were used to detect the changes of chemical groups and element composition on material surface before and after the immobilization of NPs. As shown in Figure 1(a), the peak at 1600 cm^−1^ in the SS-DA spectrum represents the C=C bond of the benzene ring, and the peak at 1502 cm^−1^ represents the *π* bond of the benzene ring. New absorption peaks appeared in the infrared spectra of CHS/Hep NP-modified samples. Among them, the peaks at 1630 cm^−1^ and 1520 cm^−1^ refer to the stretching vibrations of amide I band and amide II band. These peaks mainly derived from the amides and amines in chitosan molecules. Besides, the vibration peak of C-O-C bond can be observed at 1062 cm^−1^, which indicates the existence of heparin.

According to XPS result (Figure 1(b)), it was found that the absorption peak of S2s and S2p could be observed in the spectra of SS-DA-NP and SS-DA-NP@NGF, which demonstrated the existence of heparin. Elemental calculation results (Table 2) further proved the incorporation of NGF may significantly increase the content of N element, while the decrease of S element content may be related to the spatial shielding effect caused by the interaction between NGF and Hep. In summary, the above results show that CHS/Hep and CHS/Hep@NGF NPs are successfully immobilized on the surface of the material.

### 3.3. Morphology of NP-Modified Surface

**Figure 2:**
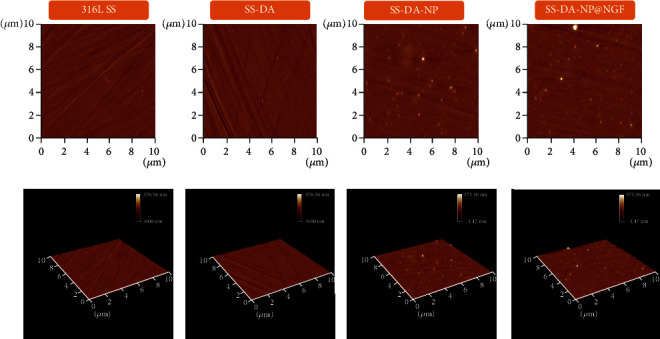
AFM images of CHS/Hep and CHS/Hep@NGF NP-modified surface.

The changes of surface morphology of different samples were detected by AFM. As shown in Figure 2, few numbers of particles could be observed on the surface of SS-DA, which may be produced during the polymerization of dopamine. A large number of nanoparticles appeared on the surface of the samples modified by CHS/Hep and CHS/Hep@NGF NPs, and the particle size was in the range of 100 to 200 nm, which was consistent with the results of particle size detection. In addition, the nanoparticles were uniformly distributed on the surface of the sample, with no obvious particle agglomeration found.

### 3.4. Surface Hydrophilicity Characterization Result

**Figure 3:**
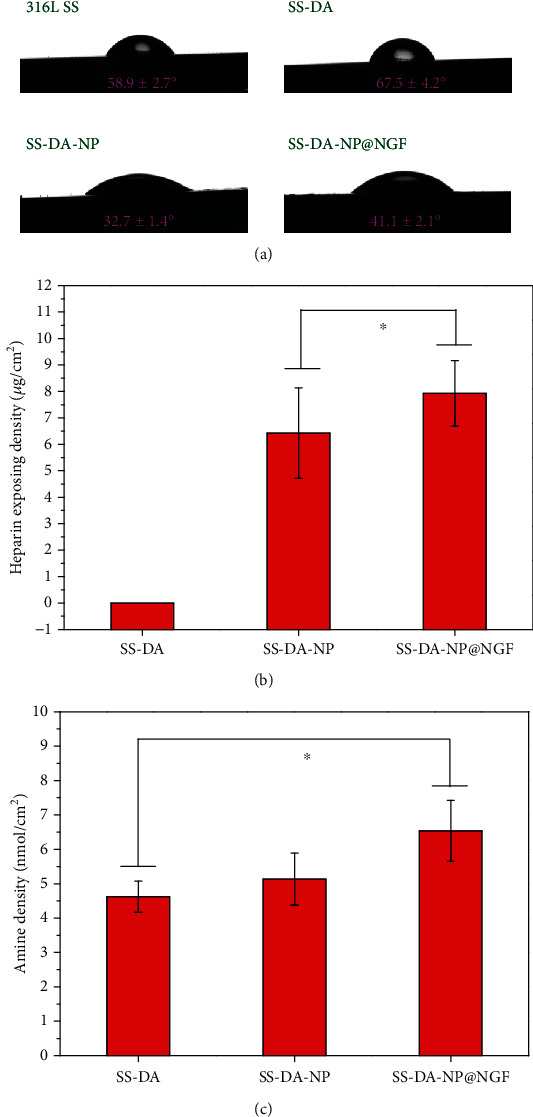
(a) Water contact angle of different sample surfaces. Quantitative characterization result of (b) heparin and (c) amine group exposing density (mean ± SD, *N* = 6, ^∗^*p* < 0.05 indicates significant difference).

As shown in Figure 3(a), the water contact angle of SS-DA was obviously increased compared with that of 316L SS, which is mainly due to the hydrophobic structure of benzene ring in dopamine coating. The immobilization of CHS/Hep nanoparticles significantly improved the surface hydrophilicity, mainly due to the rich hydrophilic groups such as amino group, carboxyl group, hydroxyl group, and sulfonic acid group in chitosan and heparin molecules. The incorporation of NGF into the nanoparticles slightly increased the water contact angle, which may be related to the exposure of hydrophobic group outside the protein structure.

The alteration of surface hydrophilicity may change the protein adsorption type and conformation and then affect its biocompatibility. In general, the surface with high hydrophobicity (water contact angle > 120°) may trigger irreversible adsorption of protein and destroy its conformation, while the surface with high hydrophilicity (water contact angle < 20°) will form water film on the surface, resulting in protein desorption [23]. In this study, the water contact angle of NP-modified surface was in the range of 40° to 60°, which may promote the adsorption of adhesive plasma proteins and thereby enhance cellular compatibility.

### 3.5. Quantitative Characterization of Heparin and Amine Group

Figure 3(b) shows the quantitative characterization results of heparin on NP-modified surface, in which SS-DA was set as blank control. According to the calculation, the exposure density of heparin on SS-DA-NP is 6.4 ± 1.7 *μ*g/cm^2^, while it slightly increased on SS-DA-NP@NGF-modified surface (7.9 ± 1.2 *μ*g/cm^2^). This may be attributed to the increase of amino group density after incorporation of NGF (Figure 3(c)), which may facilitate the covalent binding of nanoparticles to dopamine coating.

Heparin is a kind of negative-charged polysaccharide with superior anticoagulant property. According to previous study, heparin exposing density in the range of 3-7 *μ*g/cm^2^ may contribute to improving the blood compatibility of the material surface, prevent the occurrence of inflammatory reaction, and promote the healing of endothelium layer [15].

### 3.6. In Vitro Blood Compatibility Evaluation

**Figure 4:**
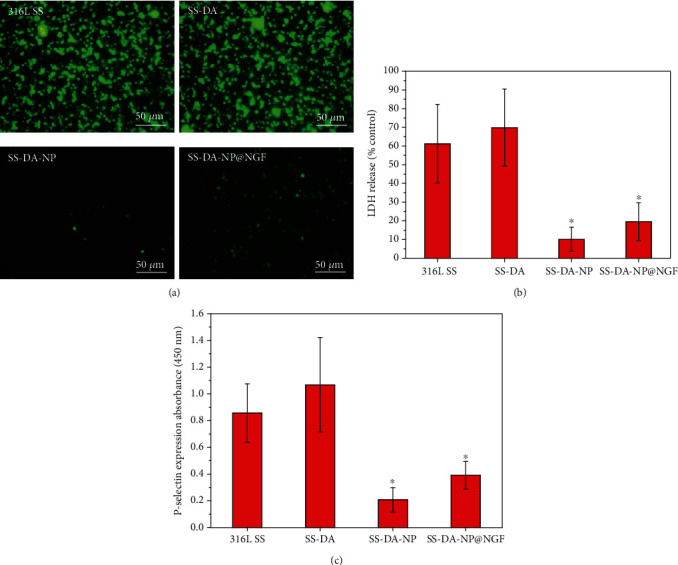
*In vitro* blood compatibility evaluation result. (a) Rhodamine 123 fluorescence staining of adherent platelets, (b) LDH release, and (c) P-selectin expression level of platelets adhered on different sample surfaces (mean ± SD, *n* = 6, ^∗^*p* < 0.05 indicates significant difference compared to 316L SS and SS-DA).

Figure 4(a) shows the fluorescence staining results of platelets adhering to the material surface for 2 hours in vitro. According to the results, a large amount of platelets was observed on the surface of 316L SS and SS-DA, and the adhered platelets were aggregated seriously, indicating serious activation and poor blood compatibility. In contrast, the adhesion density of platelets on SS-DA-NP and SS-DA-NP@NGF surface was greatly decreased, and the adherent platelets mainly displayed round shape, with almost no deformation and pseudopodium, which indicates that the nanoparticle can effectively reduce the activation of platelets and improve the hemocompatibility. The results of LDH release (Figure 4(b)) and P-selectin expression (Figure 4(c)) further proved that the surface modified by CHS/Hep nanoparticles could significantly reduce the adhesion and activation of platelet (^∗^*p* < 0.05). Although the incorporation of NGF increased the platelet adhesion density to some extent, it still showed excellent anticoagulant properties compared with 316L SS and SS-DA.

### 3.7. In Vitro Cellular Compatibility Evaluation

**Figure 5:**
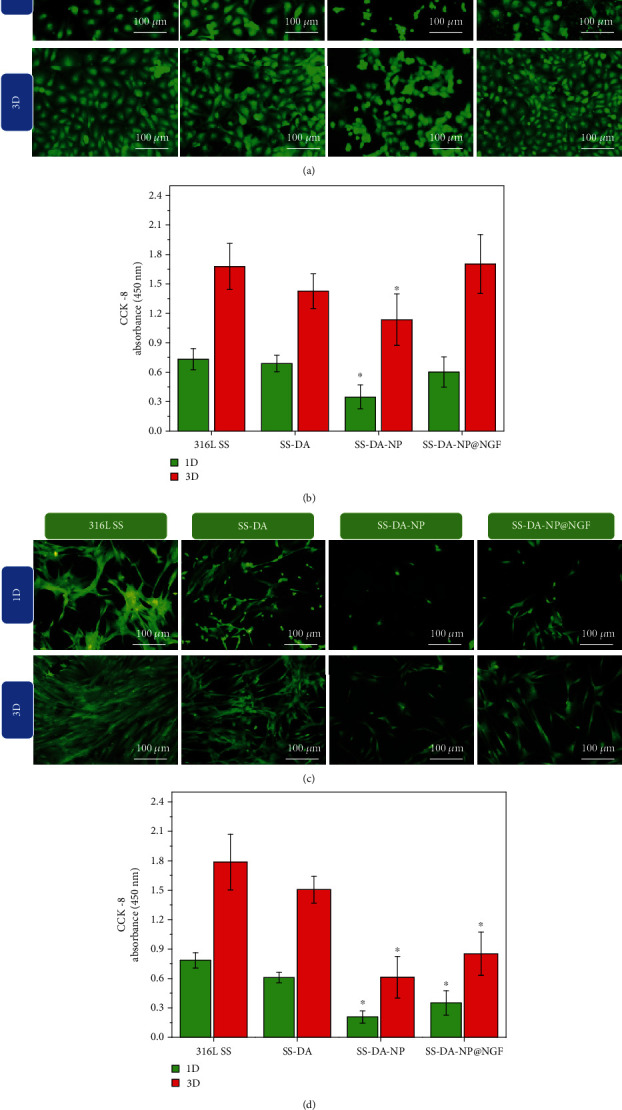
Rhodamine 123 and DAPI fluorescence staining of adhered (a) ECs and (c) SMCs after culture of 1 day and 3 days. The proliferation activity of (b) ECs and (d) SMCs was detected by CCK-8 assay (mean ± SD, *n* = 6, ^∗^*p* < 0.05 indicates significant difference compared to 316L SS and SS-DA).

Figure 5(a) shows the fluorescence staining results of vascular endothelial cells cultured on different sample surfaces for a certain period of time. The results showed that after 1 and 3 days of culture, the adherent endothelial cells on the surface of 316L SS and SS-DA showed typical cobblestone morphology, indicating that the biological function of the cells was normal. However, on SS-DA-NP surface, ECs exhibit shrinkage or round-shaped morphology, which indicated poor cell adhesion and proliferation activity. In comparison, the morphology of endothelial cells on SS-DA-NP@NGF surface was normal, and the adhesion density was similar to that of 316L SS and SS-DA. CCK-8 result (Figure 5(b)) further proved that the cell proliferation activity on SS-DA-NP surface was significantly decreased, but shows no obvious alteration on SS-DA-NP@NGF surface.

Similar to that of ECs, the adhesion density and proliferation activity of SMCs on SS-DA-NP surface was significantly decreased compared with that of 316L SS and SS-DA (Figures 5(c) and 5(d)). However, although the incorporation of NGF promotes the adhesion of the spreading of SMCs to some extent (Figure 5(c)), the proliferation activity was significantly lower (^∗^*p* < 0.05) compared with 316L SS and SS-DA (Figure 5(d)).

The results of in vitro cytocompatibility evaluation showed that the surface modified by CHS/Hep nanoparticles was not conducive to the adhesion and growth of ECs and SMCs, which was mainly related to the inhibitory effect of heparin molecules on cell proliferation [24–26]. The introduction of NGF improved the adhesion and proliferation behavior of the two kinds of cells to some extent, but the enhancement extent was different for different cells. In general, the surface of SS-DA-NP@NGF showed the effect of selectively promoting the growth of endothelial cells and inhibiting the proliferation of smooth muscle cells.

### 3.8. EPC Mobilization and Homing

**Figure 6:**
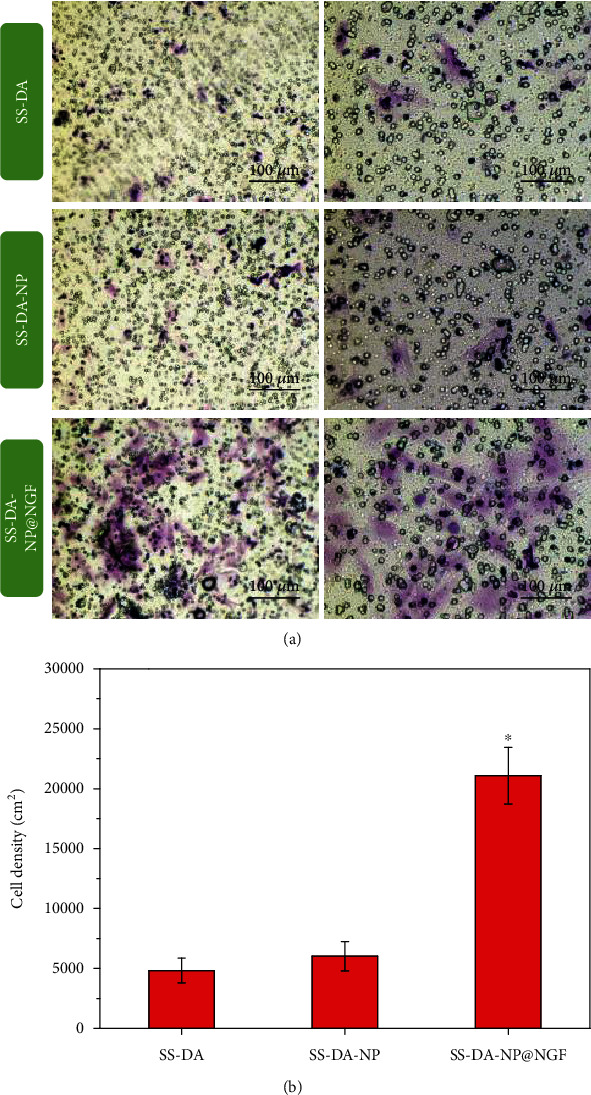
Mobilization and homing behavior of EPCs induced by nanocoating. (a) Crystal violet staining image of EPCs that mobilized to the bottom of the transwell chamber, and (b) cell counting result (mean ± SD, *n* = 6, ^∗^*p* < 0.05 indicates significant difference compared to SS-DA and SS-DA-NP).

As shown in Figure 6, SS-DA-NP@NGF has a significant chemotaxis effect on EPCs in the transwell chamber. After 24 hours of cell culture, the number of EPCs passing through the semipermeable bottom membrane to the bottom of the chamber was significantly higher than that in the SS-DA and SS-DA-NP control groups. The crystal violet staining results showed that the EPCs chemotactic by SS-DA-NP@NGF showed favorable proliferation morphology, and the cell spreading and covering area were also significantly higher than those in the control group. The results indicated that SS-DA-NP@NGF offers adequate capacity to induce the mobilization and homing of EPCs to the material surface.

In summary, since the discovery of EPCs, the mechanism of vascular endothelial injury healing has been redefined based on the migration of ECs from the surrounding vascular tissue and EPC homing at the injury site [27]. Although the surface modified by chitosan/heparin NPs shows excellent blood compatibility and the ability to inhibit the growth of smooth muscle cells, insufficient property in promoting the repair of endothelial injury continues to be problematic. NGF is a growth factor that has been reported to stimulate HUVEC proliferation and induce EPC mobilization and homing [21, 28, 29]. In this study, it was found that NGF-loaded NPs could effectively enhance the adhesion and proliferation activity of ECs on the sample surface and showed a strong chemotactic effect on vascular EPCs, indicating that the NGF-loaded nanocoating could selectively inhibit blood coagulation and intimal hyperplasia, while accelerating endothelial regeneration.

## 4. Conclusions

In this study, CHS/Hep@NGF NPs were successfully prepared by the electrostatic interaction among chitosan, heparin, and laminin and immobilized on the surface of dopamine-coated stainless steel. The results of material characterization showed that the NPs were uniformly immobilized on material surface. Biological evaluation results demonstrated that modified surface showed favorable capacity to inhibit platelet adhesion and activation and contributed to enhancing EC growth and preventing SMC overproliferation. This work has potential application for the design of polymer-free coronary artery stent coating.

## Figures and Tables

**Table 1:** Size and zeta potential of prepared nanoparticles.

	Size (nm)	Zeta potential (mV)	PDI
CHS/Hep NPs	147.8 ± 6.7	−25.6 ± 1.7	0.107 ± 0.011
CHS/Hep@NGF NPs	133.4 ± 5.5	−25.4 ± 1.2	0.069 ± 0.008

**Table 2:** Surface elemental composition determined by XPS.

Samples	C%	N%	O%	S%
316L SS	69.30	5.76	24.83	0.12
SS-DA	74.74	7.98	17.27	0
SS-DA-NP	65.78	9.68	23.44	1.11
SS-DA-NP@NGF	63.58	11.87	23.68	0.87

## Data Availability

The data used to support the findings of this study are available from the corresponding author upon request.

## References

[1] Jinnouchi H., Torii S., Sakamoto A., Kolodgie F. D., Virmani R., Finn A. V. (2019). Fully bioresorbable vascular scaffolds: lessons learned and future directions. *Nature Reviews Cardiology*.

[2] Li J. A., Chen L., Zhang X. Q., Guan S. K. (2020). Enhancing biocompatibility and corrosion resistance of biodegradable Mg-Zn-Y- Nd alloy by preparing PDA/HA coating for potential application of cardiovascular biomaterials. *Materials Science and Engineering: C*.

[3] Verheul H. M., Pinedo H. M. (2007). Possible molecular mechanisms involved in the toxicity of angiogenesis inhibition. *Nature Reviews Cancer*.

[4] Liu T., Liu S., Zhang K., Chen J. Y., Huang N. (2014). Endothelialization of implanted cardiovascular biomaterial surfaces: the development from in vitro to in vivo. *Journal of Biomedical Materials Research Part A*.

[5] Calzi S. L., Neu M. B., Shaw L. C., Kielczewski J. L., Moldovan N. I., Grant M. B. (2010). EPCs and pathological angiogenesis: when good cells go bad. *Microvascular Research*.

[6] Iivanainen E., Kahari V. M., Heino J., Elenius K. (2003). Endothelial cell-matrix interactions. *Microscopy Research and Technique*.

[7] Chiu L. L., Radisic M. (2010). Scaffolds with covalently immobilized VEGF and angiopoietin-1 for vascularization of engineered tissues. *Biomaterials*.

[8] De Visscher G., Mesure L., Meuris B., Ivanova A., Flameng W. (2012). Improved endothelialization and reduced thrombosis by coating a synthetic vascular graft with fibronectin and stem cell homing factor SDF-1*α*. *Acta Biomaterialia*.

[9] Wang J., Chen Y., Liu T. (2014). Covalent co-immobilization of heparin/laminin complex that with different concentration ratio on titanium surface for selectively direction of platelets and vascular cells behavior. *Applied Surface Science*.

[10] Zhang K., Li J. A., Deng K., Liu T., Chen J. Y., Huang N. (2013). The endothelialization and hemocompatibility of the functional multilayer on titanium surface constructed with type IV collagen and heparin. *Colloids and Surfaces. B, Biointerfaces*.

[11] Pan C. J., Hu Y. D., Hou Y. (2017). Corrosion resistance and biocompatibility of magnesium alloy modified by alkali heating treatment followed by the immobilization of poly (ethylene glycol), fibronectin and heparin. *Materials Science and Engineering: C*.

[12] Galvin P., Thompson D., Ryan K. B. (2012). Nanoparticle-based drug delivery: case studies for cancer and cardiovascular applications. *Cellular and Molecular Life Sciences*.

[13] Zhou J. L., Ding J. L., Zhu Z. G. (2019). Surface biofunctionalization of the decellularized porcine aortic valve with VEGF-loaded nanoparticles for accelerating endothelialization. *Materials Science and Engineering: C*.

[14] Mohammadi F., Golafshan N., Kharaziha M., Ashrafi A. (2019). Chitosan-heparin nanoparticle coating on anodized NiTi for improvement of blood compatibility and biocompatibility. *International Journal of Biological Macromolecules*.

[15] Liu T., Liu Y., Chen Y. (2014). Immobilization of heparin/poly-(L)-lysine nanoparticles on dopamine-coated surface to create a heparin density gradient for selective direction of platelet and vascular cells behavior. *Acta Biomaterialia*.

[16] Liu T., Zeng Z., Liu Y. (2014). Surface modification with dopamine and heparin/poly-l-lysine nanoparticles provides a favorable release behavior for the healing of vascular stent lesions. *Applied Materials & Interfaces*.

[17] Fonseca-Santos B., Chorilli M. (2017). An overview of carboxymethyl derivatives of chitosan: their use as biomaterials and drug delivery systems. *Materials Science & Engineering. C, Materials for Biological Applications*.

[18] Wang W., Xue C., Mao X. (2020). Chitosan: structural modification, biological activity and application. *International Journal of Biological Macromolecules*.

[19] Berglund J. D., Galis Z. S. (2003). Designer blood vessels and therapeutic revascularization. *British Journal of Pharmacology*.

[20] Zeng W., Yuan W., Li L. (2010). The promotion of endothelial progenitor cells recruitment by nerve growth factors in tissue-engineered blood vessels. *Biomaterials*.

[21] Jadhao C. S., Bhatwadekar A. D., Jiang Y., Boulton M. E., Steinle J. J., Grant M. B. (2012). Nerve growth factor promotes endothelial progenitor cell-mediated angiogenic responses. *Investigative Ophthalmology & Visual Science*.

[22] Liu T., Wang X., Tang X. H. (2017). Surface modification with ECM-inspired SDF-1*α*/laminin-loaded nanocoating for vascular wound healing. *ACS Applied Materials & Interfaces*.

[23] Monchaux E., Vermette P. (2010). Effects of surface properties and bioactivation of biomaterials on endothelial cells. *Frontiers in Bioscience (Scholar Edition)*.

[24] Letourneur D., Caleb B. L., Castellot J. J. (1995). Heparin binding, internalization, and metabolism in vascular smooth muscle cells: I. Upregulation of heparin binding correlates with antiproliferative activity. *Journal of Cellular and Comparative Physiology*.

[25] Ettelaie C., Fountain D., Collier M. E. W., Elkeeb A. M., Xiao Y. P., Maraveyas A. (2011). Low molecular weight heparin downregulates tissue factor expression and activity by modulating growth factor receptor-mediated induction of nuclear factor-*κ*B. *Biochimica et Biophysica Acta*.

[26] Li L., Rui X., Liu T. F., Xu G. L., He S. Y. (2012). Effect of heparin-derived oligosaccharide on vascular smooth muscle cell proliferation and the signal transduction mechanisms involved. *Cardiovascular Drugs and Therapy*.

[27] Avci-Adali M., Ziemer G., Wendel H. P. (2010). Induction of EPC homing on biofunctionalized vascular grafts for rapid in vivo self-endothelialization -- a review of current strategies. *Biotechnology Advances*.

[28] Yu X., Qi Y., Zhao T. (2019). NGF increases FGF2 expression and promotes endothelial cell migration and tube formation through PI3K/Akt and ERK/MAPK pathways in human chondrocytes. *Osteoarthritis and Cartilage*.

[29] Moser K. V., Reindl M., Blasig I., Humpel C. (2004). Brain capillary endothelial cells proliferate in response to NGF, express NGF receptors and secrete NGF after inflammation. *Brain Research*.

